# HIV/AIDS and lipodystrophy: Implications for clinical management in resource-limited settings

**DOI:** 10.7448/IAS.18.1.19033

**Published:** 2015-01-15

**Authors:** Julia L Finkelstein, Pooja Gala, Rosemary Rochford, Marshall J Glesby, Saurabh Mehta

**Affiliations:** 1 Division of Nutritional Sciences, Cornell University Ithaca, NY, USA; 2 St. John's Research Institute, Bangalore, India; 3 Division of Infectious Diseases, Weill Cornell Medical College, New York, NY, USA; 4 Department of Microbiology and Immunology, SUNY Upstate Medical University, Syracuse, NY, USA

**Keywords:** HIV, AIDS, lipodystrophy, fat redistribution, antiretroviral therapy

## Abstract

**Introduction:**

Lipodystrophy is a term used to describe a metabolic complication of fat loss, fat gain, or a combination of fat loss and gain, which is associated with some antiretroviral (ARV) therapies given to HIV-infected individuals. There is limited research on lipodystrophy in low- and middle-income countries, despite accounting for more than 95% of the burden of HIV/AIDS. The objective of this review was to evaluate the prevalence, pathogenesis and prognosis of HIV-related lipoatrophy, lipohypertrophy and mixed syndrome, to inform clinical management in resource-limited settings.

**Methods:**

We conducted a structured literature search using MEDLINE electronic databases. Relevant MeSH terms were used to identify published human studies on HIV and lipoatrophy, lipohypertrophy, or mixed syndrome in low-, low-middle- and upper-middle-income countries through 31 March 2014. The search resulted in 5296 articles; after 1599 studies were excluded (958 reviews, 641 non-human), 3697 studies were extracted for further review. After excluding studies conducted in high-income settings (*n*=2808), and studies that did not meet inclusion criteria (*n*=799), 90 studies were included in this review.

**Results and Discussion:**

Of the 90 studies included in this review, only six were from low-income countries and eight were from lower middle-income economies. These studies focused on lipodystrophy prevalence, risk factors and side effects of antiretroviral therapy (ART). In most studies, lipodystrophy developed after the first six months of therapy, particularly with the use of stavudine. Lipodystrophy is associated with increased risk of cardiometabolic complications. This is disconcerting and anticipated to increase, given the rapid scale-up of ART worldwide, the increasing number and lifespan of HIV-infected patients on long-term therapy, and the emergence of obesity and non-communicable diseases in settings with extensive HIV burden.

**Conclusions:**

Lipodystrophy is common in resource-limited settings, and has considerable implications for risk of metabolic diseases, quality of life and adherence. Comprehensive evidence-based interventions are urgently needed to reduce the burden of HIV and lipodystrophy, and inform clinical management in resource-limited settings.

## Introduction

Since the introduction of highly active antiretroviral therapy (HAART) as a treatment for HIV and AIDS, HIV-related mortality has been reduced by 50 to 80% [1]. However, HIV-infected individuals on long-term therapy are at increased risk for developing a variety of metabolic disturbances, including fat redistribution, dyslipidemia, insulin resistance, lactic acidemia and abnormalities in bone mineral metabolism, particularly with nucleoside reverse transcriptase inhibitors (NRTIs) and protease inhibitors (PIs) [2–6]. Several of these complications, particularly lipodystrophy, can predispose individuals to cardiovascular disease and impact quality of life and adherence to antiretroviral therapy (ART) [5,7–9].

Research on metabolic complications of ART has been primarily conducted in high-income countries. There is increasing evidence from low- and middle-income countries evaluating the short- and long-term effects of ART on lipodystrophy syndromes such as lipoatrophy, lipohypertrophy and mixed syndrome. *Lipoatrophy* is the loss of subcutaneous fat, particularly evident in the face, buttocks and limbs. *Lipohypertrophy* is the accumulation of visceral and central fat in the abdomen, dorsocervical region (“buffalo hump”), or breasts. *Fat redistribution* is an all-encompassing terms used to describe lipohypertrophy, lipoatrophy and/or mixed syndrome [10–12]. In early studies, lipodystrophy was used to describe a syndrome of subcutaneous fat loss with or without visceral fat accumulation, insulin resistance and/or dyslipidemia [13]. Although most studies to date use “lipodystrophy” as an all-encompassing term for fat redistribution, current research emphasizes the need to differentiate isolated lipoatrophy, lipohypertrophy and mixed syndrome as separate disease processes [13–15]. In this review, fat redistribution syndromes will be defined based on clinical guidelines and as defined by study authors.

In the context of rapid ART scale-up in resource-limited settings, addressing lipodystrophy is a critical component of HIV/AIDS care and treatment. The antiretroviral (ARV) medications linked to lipodystrophy, particularly lipoatrophy, are prevalent in low-income and middle-income settings [16], where HIV-infected populations face the dual burden of obesity and undernutrition [17,18]. There is a need to evaluate the prevalence, pathogenesis and prognosis of lipoatrophy, lipohypertrophy and mixed syndrome, to inform clinical management in low- and middle-income countries.

## Methods

### Search strategy

**Figure 1:**
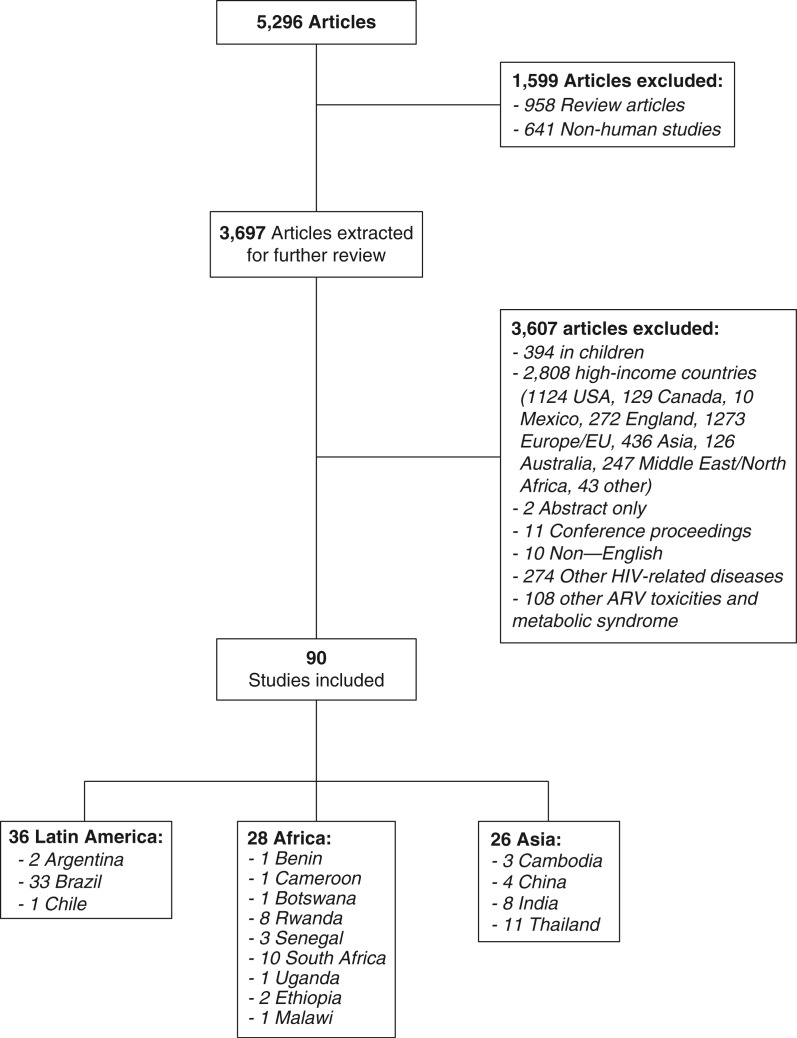
Search strategy.

We conducted a structured literature search using MEDLINE electronic databases to identifiy published studies through 31 March 2014. Search terms included: (HIV [MH] OR HIV Infect*[MH] OR Human Immunodeficiency Virus [tw] OR Human Immuno-deficiency Virus [tw] OR hiv-1 [tw] OR hiv-2 [tw] OR hiv*[tw] OR AIDS [MH] OR AIDS [tw]) AND (Lipodystrophy [MH] OR Lipoatrophy [MH] OR -hypertrophy [MH] OR buffalo hump [tw] OR lipo-accumulation [tw] OR lipo*[MH] OR adiposity [tw] OR Lipodystrophy [tw] OR Lipoatrophy [tw] OR Lipohypertrophy [tw] or Fat distrib*[tw] or Fat redistrib*[tw] OR fat accumulation [tw] or lipo-*[tw]) [19]. The search strategy and results are summarized in Figure 1, and findings from these studies are summarized in detail in the supplementary tables (Tables 1–3).

The structured literature search resulted in 5296 articles; after 1599 studies were excluded (958 reviews, 641 non-human), 3697 studies were extracted for further review. Sources were retrieved, collected, indexed and assessed for HIV- and lipodystrophy-related data. The inclusion criteria for this review were availability of data on human HIV-related fat redistribution (lipodystrophy, lipoatrophy, fat redistribution, lipo-accumulation and/or lipohypertrophy), abstract availability, age ≥18 years, and low-, low-middle- and upper-middle-income countries, as defined by the World Bank classification [20].

After excluding studies conducted in high-income settings (*n*=2808), and studies that did not meet inclusion criteria (*n*=799), 90 studies were included in this review. These included 28 from Africa, 26 from Asia and 36 from Latin America.

## Literature review

### Prevalence of lipodystrophy

The global prevalence of lipodystrophy among HIV-infected adults on ART ranges considerably, from less than 1 to 84% [4,12,21–41]. The burden of HIV-related lipodystrophy is estimated to be high in low- and middle-income countries [4,12,24–41], with an incidence of 1.4 to 20.6 per 100 person-years [33,42,43], and 6.7 to 22.1 per 100 person-years [27,32], in studies in Asia and Africa, respectively. The wide ranges in prevalence reflect variations in study design, gender balance in studies, types of ARVs and duration of therapy. Understanding the major risks factors for lipodystrophy, lipoatrophy, lipohypertrophy and mixed syndrome may help explain the wide range in prevalence around the world and inform prevention and clinical management. Lipodystrophy may develop within four to six months of ART initiation, and increases considerably after 12 months [11,32,44]. For example, the prevalence of lipodystrophy was 0.8% in a study in Thailand after six months of ART [32], 34% in a study in Rwanda after 16 months of ART [11], and 63% in a study in Cambodia after four years of ART [44]. However, this trend has not been observed in all studies, and newer studies indicate that it is actually isolated lipoatrophy, not lipohypertrophy that progresses over time with tNRTIs (thymidine-based) [13,45]. In contrast, some cohort studies have noted that lipoatrophy persists but does not increase with time [46], while some trials have demonstrated that ARVs may be associated with increases in limb fat over time [47].

In a South African study in 2670 HIV-infected adults initiating HAART, the incidence of lipodystrophy was 1.4 per 100 person-years, but nearly doubled to 2.6 per 100 person-years when the follow-up period excluded the first six months of therapy [48]. Thus, ART type and duration need to be considered when comparing rates of lipodystrophy across studies. Some studies have reported the prevalence of lipoatrophy, lipohypertrophy and a mixed syndrome separately, and are presented in Table 1 [11,12,33,49–51].

**Table 1:** Prevalence of lipohypertrophy, lipoatrophy and mixed syndrome in low and middle-income countries versus high-income countries

Lipodystrophy	Africa [11,33,34,43,49]	Asia [31,32,50,51,161]	Latin America [39,162]	High-income [52,124,163,164]
Lipohypertrophy (%)	4.9–24.1	3.6–53	15.0–43.0	7.4–47.0
Lipoatrophy (%)	2.0–9.8	0.8–72.4	17.3–20.7	16.4–43.0
Mixed Syndrome (%)	2.5–33.0	22.7–26.8	34.0–46.7	12.0–33.0

In a study of 180 HIV-infected adults from Brazil, sex-specific differences in the prevalence of different lipodystrophy syndromes were observed. Women were more likely to develop lipohypertrophy (43%) or mixed syndrome (40%), while men presented with a similar prevalence for all three lipodystrophy syndromes (34% lipoatrophy, 32% lipohypertrophy, 34% mixed syndrome) [39]. The propensity of women to lipohypertrophy has also been observed in high-income countries [52].

**Figure 2:**
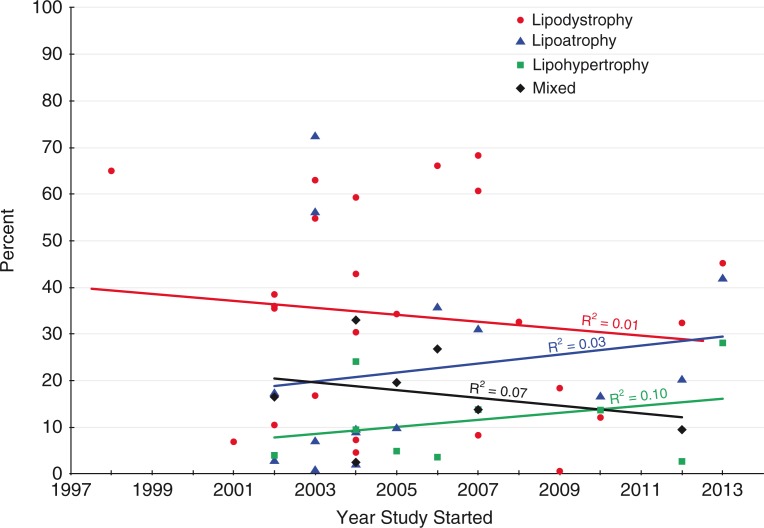
Prevalence of lipodystrophy by year study started.

In contrast to lower-income countries, the prevalence of lipoatrophy is decreasing in high-income countries, as older drug regimens are being replaced with less toxic alternatives. For example, in the Swiss Cohort Study, there was a reduced risk of lipodystrophy in 6016 HIV-infected patients who initiated ART between 2003 and 2006 compared to the 5777 who started ART between 2000 and 2002 (*p*=0.02) [22,53]. Zidovudine and stavudine use declined considerably during this period, from 88.5% and 4.2% (2000–2002) to 64.2% and 0.7% (2003–2006), respectively [22]. This does not appear to be the case in the countries included in this review (Figure 2).

The risk factors for fat redistribution syndromes and their relative importance also vary in different settings. For example, stavudine was used as standard therapy in Thailand as part of the GPO-VIR regimen [28,29]. However it has not been used as frequently in Brazil, affecting the prevalence of lipoatrophy [54–59] as seen in Table 1.

When comparing prevalence of lipodystrophy among studies, country-specific drug regimens, gender proportions and duration of ART use should also be considered.

### Diagnosing lipodystrophy, lipoatrophy and lipohypertrophy

Fat redistribution can be difficult to diagnose, particularly in settings where populations face the dual burden of obesity and undernutrition [17,18]. There is no universal definition for lipodystrophy or body fat redistribution; the term “lipodystrophy” is often used to categorize any form of fat redistribution [60]. As a result, the diagnosis, clinical management, and assessment of prevalence and aetiology are challenging. There is also a lack of concordance in the methods used to diagnose and monitor fat redistribution.

Lipodystrophy assessment methods include clinical examination, self-report, anthropometry, bioelectrical impedance analysis (BIA) and imaging techniques such as dual-energy X-ray absorptiometry (DEXA), magnetic resonance imaging (MRI) and computed tomography (CT).

#### Anthropometry

Anthropometric measurements are the most common method of diagnosing lipodystrophy and include height, weight, body mass index (BMI), mid-upper arm circumference, waist circumference, hip circumference, waist-to-hip ratio and skinfold thickness measurements. These methods are non-invasive, relatively low-cost and interpretable, and compare well with more advanced techniques of assessing lipodystrophy, such as DEXA or MRI [61–63]. However, accurate and precise measurements require careful training and ongoing supervision of field workers [64].

Standardized techniques are used to assess *skinfold thickness measurements*, including tricipital, bicipital, subscapular and suprailiac folds. The Durnin-Wormersley equation is often used to calculate body fat percentage from skinfold thickness measurements and has been validated in different populations, including African-Americans in the United States [65–67]. These tools are feasible and widely used in resource-limited settings [68,69]. However, these equations have not been validated in HIV-infected populations, or in the context of lipodystrophy [70,71].

#### Standardized scales

The Carr Lipodystrophy Case Definition (LDCD) and the Lichtenstein HIV Outpatient Scale (HOPS) are standardized scales that are commonly used to assess lipodystrophy. LDCD uses a combination of demographic (age) and clinical (duration of HIV infection) information, metabolic parameters (anion gap, serum HDL cholesterol), anthropometry (waist-to-hip ratio) and radiologic assessments (DEXA, CT) to define lipodystrophy with 79% sensitivity and 80% specificity [60]. The LDCD has also been expanded to include a scale for lipodystrophy severity [72]. The required use of DEXA or CT limits the applicability of LDCD in resource-limited and field settings. The Lichtenstein HOPS scale was designed to assess lipodystrophy severity using standardized clinical evaluations, laboratory values and anthropometric measurements. It uses a more specific definition, including only moderate to severe lipodystrophy [73]. However, the LDCD and HOPS do not differentiate among the three lipodystrophy syndromes.

Two studies from Africa demonstrated the importance of a standardized definition for different lipodystrophy syndromes. In Senegal, 180 HIV-infected adults on HAART for four to nine years were evaluated for lipodystrophy [12]. The prevalence of mild to severe lipodystrophy diagnosed by physicians trained to evaluate lipodystrophy, lipohypertrophy and lipoatrophy using the Carr criteria was 65% (35.6% lipohypertrophy). The prevalence was 31% (14.5% lipohypertrophy) using a stricter definition which included only moderate to severe lipodystrophy, and 50% (no separate criteria for lipohypertrophy) using objective metabolic and anthropometric criteria [12]. In a study in Cameroon, the prevalence of mild to severe lipodystrophy was 60%, compared to 18% using a stricter definition that only included moderate to severe lipodystrophy [36]. In addition, a lack of concordance between clinical diagnosis and patient self-report of lipodystrophy frequently occurs, as seen in a study of 72 HIV-infected patients on HAART from Argentina. In this study, the use of two different patient questionnaires resulted in a diagnosis of body fat redistribution of 49% and 86%. When two different clinical criteria were used, the diagnosis of body fat redistribution was considerably lower, or 24% and 48%, respectively [74].

#### Body composition

Assessment is particularly important in HIV-infected individuals, since both HIV and ART are associated with changes in body composition and metabolic complications [75,76]. Weight and BMI are crude measures of body composition, particularly at the individual level. However, some methods to assess body composition, such as DEXA, are prohibitively expensive and challenging to implement in resource-limited settings.

#### Bioelectrical impedance

BIA is a non-invasive method of body composition assessment, which is more precise than skinfold thickness [70]. The use of BIA has become widespread in body composition research due to its ease of use, portability and relatively low cost. BIA indirectly estimates different body tissues and compartments based on their conduction of a low-voltage, alternating current [77]. BIA can be used to estimate total body water, fat-free body mass and body fat. BIA is useful in monitoring body changes over time and is highly correlated with other radiologic measures of body fat [70]. However, the calculation of total fat is affected by fat distribution, making it a less accurate tool for evaluation of lipodystrophy [71]. Some data suggest that BIA may not accurately reflect body composition changes in the context of HIV infection [77], and BIA has not been extensively validated in the ART era.

#### Other methods

Additional methods for assessment of body composition include imaging techniques such as DEXA, MRI and CT. However, DEXA cannot differentiate between subcutaneous and visceral fat, or evaluate facial lipoatrophy, one of the more disturbing morphologic complications of ART reported by patients [78,79]. The error in measurement of body composition, particularly central fat, using DEXA may also be higher in the context of obesity [80], suggesting that its applications may be limited in the context of lipohypertrophy assessment. In contrast to DEXA, which is unable to differentiate type of fat, MRI techniques require longer imaging time and specialized equipment, and CT has improved resolution but necessitates greater ionizing radiation exposure [80]. Additional methods, such as MRI, are considered superior in assessment of body composition in the context of HIV, though are less widely available in lower-income settings. Other methods, such as ultrasound, have not been validated for lipodystrophy assessment [81], and vary considerably on the operator [82].

In a pilot study in Canada comparing BIA, skinfold measurements, and DEXA in assessment of percent body fat mass among 47 HIV-infected men receiving HAART [83], the level of error and bias in BIA and skinfold methods were relatively low, compared to DEXA. Authors concluded that BIA and skinfold techniques are acceptable methods of monitoring total body fat mass in HIV-infected men on HAART as long as training and technique are standardized.

### Lipodystrophy pathogenesis in HIV

Lipodystrophy has a multi-factorial etiology, including HIV, effects of ART and host factors. Furthermore, lipoatrophy and lipohypertrophy likely have different pathogenic and genetic mechanisms, as reviewed elsewhere [84,85], which pose challenges to clinical diagnosis and management. HIV infection may contribute to fat redistribution by infecting macrophages in adipose tissue, which release pro-inflammatory cytokines and enhance local inflammation [86]. This is supported by increased tumour necrosis factor-alpha (TNF-α) expression observed in ART-naïve HIV-infected patients, a pro-inflammatory cytokine that initiates adipocyte apoptosis [86,87].

Although the risk of developing lipodystrophy was thought to be associated with the type and duration of ART, as seen in many studies reviewed, this inadequately describes the association between ARTs and different lipodystrophy syndromes. *In vitro* and *in vivo* research has demonstrated effects of ARVs on functions of various organs, including adipose, liver and muscle. Lipodystrophy may develop as a result of the effects of specific NRTIs and PIs on lipid metabolism [88,89]. PIs and NRTIs have been shown to alter regulation of genes involved in adipocyte differentiation, metabolism, cell cycle control and apoptosis. PIs are linked with adipocyte toxicity through several potential mechanisms [90]. PIs induce or up-regulate genes that inhibit adipocyte differentiation and down-regulate adipogenesis-related transcription factors [91,92], including interference with essential cellular transcription factors (e.g. SREBP-1c) [92]. PIs inhibit pre-adipocyte differentiation by up-regulating the Wingless-related integration site (wnt)/B-catenin signalling pathway. One of the functions of this pathway is to inhibit adipogenic gene expression. Specifically, activation of the wnt/B-catenin signalling pathway prevents the induction of PPAR-γ [93,94], a nuclear-receptor which regulators adipocyte differentiation and maintenance [95]. PIs are also linked to reduced lipid accumulation in adipocytes, increased adipocyte apoptosis and induction of insulin resistance [91].

NRTIs, stavudine and didanosine, are linked to inhibition of mitochondrial RNA transcription, depletion of mitochondrial DNA (mtDNA) and mitochondrial dysfunction, *via* inhibition of DNA polymerase γ [96]. The toxic effects of NRTIs on mitochondrial function are likely a major contributor to the development of lipoatrophy [96–99]. NRTIs inhibit mtDNA *in vitro* with varying affinity, as per below:

zalcitabine>didanosine>stavudine>zidovudine >lamivudine=abacavir=tenofovir [100]

*Most potent mtDNA inhibition to Least potent mtDNA inhibition*

Previous studies found that HIV-infected adults are less likely to develop lipoatrophy on tenofovir compared to stavudine, and tenofovir improved lipoatrophy in adults who previously developed lipoatrophy on stavudine [101,102]. These findings are of particular relevance to resource-limited settings where older NRTIs (e.g. stavudine) are still relatively common [103].

### Natural history of lipodystrophy

Lipodystrophy is often measured as a dichotomous outcome at a single time point in studies, rather than as a continuum of fat redistribution assessed prospectively. HIV-infected adults initiating ART often experience an increase in weight, trunk and limb fat during the first four to six months of therapy; after this point, trunk fat stabilizes and limb fat progressively decreases [104,105]. This may be due to an initial period of immune reconstitution, followed by NRTI or PI dose-dependent adipocyte toxicity. In a study in Rwanda, participants taking stavudine-based HAART experienced a rapid increase in body weight during the first 6 to 12 months of treatment, followed by a progressive decline in body weight during the second year of therapy, with a median loss of 3.1 kg per year (IQR: −1.6 to −5.6; *p*<0.01) [106]. In a study from Uganda, during the first six months of primarily AZT-based ART initiated by 76 HIV-infected adults, total lean and fat mass, and arm, waist, hip and thigh circumferences significantly increased [107]. The total lean mass plateaued 6 to 12 months post-ART initiation at an increased level compared to at the initiation of AZT-based ART. Abdomen, subscapular and thigh skinfold thicknesses continued to increase significantly [107].

Lipoatrophy may be associated with overall weight loss in HIV-infected adults on ART. In a study of 705 HIV-infected patients initiating stavudine in Rwanda, lipoatrophy was associated with a 2.0 kg per year weight loss (95% CI: −0.6 to −3.4 kg; *p*<0.01). Weight loss had a positive predictive value for lipoatrophy of 21% and 32% and a negative predictive value of 99% and 92% for men and women, respectively [106]. In contrast, some research suggests that lipohypertrophy presents with physiologic processes similar to regaining weight after starvation, as seen in patients recovering from *anorexia nervosa* [108]. Thus, this may indicate a restoration to health, rather than a disease process [109].

Differentiating between HIV itself, immune reconstitution and ART toxicity in the aetiology of lipodystrophy needs to be further elucidated. Prospective studies are needed to examine changes in fat redistribution over time and progression of lipodystrophy, to inform prevention and clinical management of lipodystrophy in HIV-infected patients.

### Risk factors of lipodystrophy

Risk factors for lipodystrophy syndromes identified in studies from lower-income countries are summarized in Table 2. Stavudine use, ART duration and female sex were risk factors for lipodystrophy and lipoatrophy. There were limited studies on lipohypertrophy and no risk factors were identified for mixed syndrome.

**Table 2:** Risk factors for lipodystrophy identified in studies from low- and middle-income countries

Variables	Lipodystrophy^a^	Lipoatrophy	Lipohypertrophy
ARV	Stavudine use [12,50]	Stavudine use [11,42,62]	
		Indinavir use [62]	
	Duration of ART [29,34,48]	Duration of ART [11]	
Demographic	Female sex [27,29,32,48,49,51]	Female sex [11,32,42,43,49,106]	
		Age >40 years [32]	Younger age [33]
Nutritional		Baseline BMI >25 kg/m^2^ [11]	
		Onset and rate of weight loss [11]	
Immune	Undetectable HIV RNA [50]		

a These studies identified risk factors for lipodystrophy and did not differentiate between types of lipodystrophy syndromes (i.e. lipoatrophy, lipohypertrophy and/or mixed syndrome). No risk factors were identified for mixed syndrome alone.

#### Female sex

Several studies in lower-income countries have identified female sex as a risk factor for lipodystrophy. In a study of 2190 Rwandan HIV-infected patients initiating HAART, women had a 9.7 (HR: 9.7, 95% CI: 4.5–21.0, *p*<0.05) times greater risk of developing lipoatrophy, compared to men [43]. In a study in South Africa, the prevalence of lipoatrophy was 57% in women, compared to 13% in men (*p*<0.05) [49]. In some studies, female sex was the only significant risk factor for lipodystrophy, after adjusting for other variables [42,49,106]. Women may also be less likely to receive adequate health care, education and support for HIV/AIDS [8,110] and may be more vulnerable to the social impact of stigma and metabolic consequences [8,110].

#### Stavudine treatment

Stavudine is associated with severe dose-related side-effects of lipoatrophy, lactic acidosis and peripheral neuropathy [32,48,99,111]. Based on these findings, the World Health Organization (WHO) recommended a lower maximum stavudine dose for all adults in 2007 (40–30 mg) [36,51,112], and discontinued use in 2009 [113]. Despite WHO recommendations, 14 (of 52) developing countries still used stavudine as a first-line HIV therapy in 2010, due to its comparatively lower cost (~18 USD/year) and widespread availability [16,114,115].

Stavudine toxicity continues to be a common cause of metabolic complications and regimen changes in resource-limited settings. HIV-infected patients taking stavudine had a 2.8 (OR: 2.8, 95% CI: 1.4–5.5, *p*<0.05; OR: 4.7, 95% CI: 1.3–17.1, *p*=0.019) [11,12] to 7.4 (OR: 7.4, 95% CI: 1.3–40.8, *p*=0.022) [62] times greater odds of lipoatrophy, compared to other NRTI and ARV regimens. In Rwanda, the prevalence of lipodystrophy was three times higher in HIV-infected patients on stavudine, compared to zidovudine, which also causes lipoatrophy [11]. In Cameroon, HIV-infected patients on stavudine were 5.5 times as likely to have lipoatrophy, compared to zidovudine (RR: 5.5, 95% CI: 1.3–23.5, *p*=0.02) [36,116]. Stavudine toxicity also increases with duration and dose [117]. In a cross-sectional study of 103 HIV-infected adults in Thailand, stavudine duration was associated with increased odds of lipodystrophy, with a 2% higher odds per month of treatment [117]. Despite WHO recommendations and scientific evidence on its severe side-effects, however, its lower price and widespread availability makes stavudine replacement challenging in lower-income countries.

#### Protease inhibitors

PIs were originally thought to be the main causative agent of lipodystrophy, though more recent evidence identified thymidine NRTIs as the leading contributor to ARV-related lipodystrophy. Lipohypertrophy has been attributed to PI use [14,118].

There is limited research on PIs in lower-income countries, due in part to their prohibitive cost, compared to NRTIs and non-nucleoside reverse transcriptase inhibitors (NNRTIs) [114,119]. PIs are widely used in upper-middle-income countries [30,62,120–122]. In this review, studies in only three countries – Thailand, India and Brazil – evaluated the effects of PI use on lipodystrophy. In Thailand, 247 HIV-infected adults taking PIs had a higher prevalence of facial atrophy (50% vs. 30%) and female breast hypertrophy (52% vs. 12%), compared to non-PI ART [62]. In India, patients who switched to a PI after immunologic failure on NNRTIs had a 10% incidence of lipodystrophy within the first year [30]. In a cross-sectional study of 457 HIV-infected adults and adolescents in Brazil, longer PI use was associated with self-reported lipodystrophy [122].

#### NNRTIs

NNRTIs, particularly nevirapine, are used frequently in lower-income countries and are not commonly linked to lipodystrophy syndromes. In one study, nevirapine was associated with a 50% lower odds of lipoatrophy (OR 0.50, 95% CI: 0.26–0.95, *p*<0.001) [62].

#### Other risk factors

Additional risk factors for lipodystrophy identified in the studies reviewed include: older age (>40 years) [32,73,123,124], greater baseline body weight at ARV initiation [29,48], undetectable HIV RNA [50], recent weight loss and faster rate of weight loss [11]. Increased baseline weight and BMI are risk factors that should be carefully examined in settings characterized by concurrent obesity and undernutrition.

### Implications of lipodystrophy

The development of lipodystrophy is associated with cardiometabolic risk factors. In a study of 68 underweight (BMI <18.0 kg/m^2^) HIV-infected adults initiating NRTIs in India, 19 to 25% (Diabetes Federation criteria versus National Cholesterol Education Program criteria) of participants developed new onset metabolic syndrome within six months of ART initiation [125]. Patients with ART-induced lipodystrophy may also develop impaired glucose tolerance, insulin resistance, hyperlactatemia, dyslipidemia and increased inflammatory markers [126,127]. Although the interrelationship between ART use, insulin resistance and lipodystrophy has been noted in several studies [127–129], the temporal relationship and biological mechanisms involved are not well-understood. Insulin resistance often precedes lipodystrophy, so it is thought to be involved in the pathogenesis and development of lipodystrophy [127]; however, other studies have suggested that lipodystrophy contributes to the development of insulin resistance [128,129].

Lipodystrophy is associated with a variety of metabolic disturbances, though the causal mechanisms are unknown [50]. In a study in Thailand, 93% of HIV-infected patients with lipodystrophy had at least one metabolic abnormality [130]. Lipodystrophy has been associated with increased triglycerides [27], total cholesterol [34,130] and elevated glucose [130], but no associations have been found with HDL or LDL [27]. Sex differences have been observed in the presentation of distinct metabolic risk profiles. For example, in a prospective study of 332 HIV-infected adults in Brazil, BMI was higher in HIV-infected women with lipodystrophy (*p*=0.001) but lower in HIV-infected men with lipodystrophy (*p*=0.04) [37]. In contrast, HIV-infected men with lipodystrophy had lower HDL, compared to women [37].

#### Quality of life and ARV adherence

Lipodystrophy is also associated with psychological consequences [131,132], reduced self-reported quality of life and ARV adherence [8,59]. In a cross-sectional study in Thailand, 278 HIV-infected adults ranked the perceived severity of their lipodystrophy as mild (28%), moderate (54%) and severe (15%) [130]. Self-reported lipodystrophy correlated poorly with clinical diagnosis of lipodystrophy (60% mild, 20% moderate, 2% severe) [130]. Lipodystrophy has also been associated with lower reported quality of life. Patients have described the effects of lipodystrophy as disturbing and reported reduced satisfaction with their body image, self-esteem and social relationships, less confidence about their health and embarrassment due to body changes [8,11,133]. These negative psychosocial consequences can also be associated with a fear of disclosure of HIV status and of stigma associated with HIV/AIDS. In a study of 50 adults in South India, there was a significant decrease in quality of life regarding financial (*p*<0.045) and disclosure (*p*<0.032) concerns between patients with lipodystrophy and without lipodystrophy [134]. In a study in patients with lipodystrophy in Thailand, despite improvements seen on DEXA (after stavudine/didanosine substitution with tenofovir/lamivudine), there were no significant changes in patients’ perceptions of their own lipodystrophy [99]. In addition to the physical consequences of lipodystrophy, evaluating the impact of lipodystrophy on emotional well-being and quality of life is an important component to clinical management.

### Management and treatment of lipodystrophy

The primary therapy for severe lipodystrophy, particularly lipoatrophy, is a change in ARV regimen. There are also an increasing number of clinical management and treatment options for lipoatrophy and lipohypertrophy in high-income countries. However, there is comparatively little research on clinical management and treatment of lipodystrophy in lower-income countries.

#### Lifestyle modifications

Diet and exercise can help reduce the progression of lipodystrophy, and mitigate its impact on health and quality of life. In a study in Rwanda, among HIV-infected individuals with lipodystrophy, a six-month exercise programme improved cardiorespiratory fitness, metabolic parameters and quality of life, compared to individuals not enrolled in exercise therapy [133,135]. Participants also experienced a reduction in total cholesterol and insulin levels and improved body image, self-esteem, psychological well-being and social relationships [133,135]. Several studies from Brazil have reported similar effects of lifestyle programmes [136,137]. Further research is needed to determine the impact of exercise programmes on the treatment of lipodystrophy, and particularly on severe lipoatrophy, as exercise may exacerbate this condition [138,139]. In contrast, in a study from Brazil where an intervention group received diet counselling every two months, there were no differences in anthropometric measurements between the intervention and control groups [140].

#### Dose reduction

In 2007, the WHO recommended a stavudine dose reduction from 40 to 30 mg for adults and adolescents [36]. During this time period, researchers in Cameroon and South Africa monitored changes in lipodystrophy and metabolic parameters [36,116]. In both cohort studies, the prevalence of lipoatrophy was significantly higher in patients who had taken the 40 mg dose of stavudine, compared to those who had only taken the 30-mg dose [36,116]. In the cohort study in South Africa among 249 HIV-infected adults, patients taking the 40 mg dose of stavudine had a 12 times greater odds of moderate to severe lipoatrophy (OR: 11.8, 95% CI: 3.2–43.8, *p*<0.05), compared to 30 mg [116]. In a meta-analysis of trials comparing these doses of stavudine in high-income countries, there was strong evidence that the lower 30 mg dose of stavudine was associated with lower rates of side-effects (e.g. peripheral neuropathy) and discontinuation, and similar efficacy in suppressing HIV viral load, compared to the 40 mg dose. These findings informed the WHO recommendations for lower dose stavudine in 2007 [141] and the subsequent discontinuation of stavudine due to severe side-effects in 2009 [113].

#### Regimen switches

Lipodystrophy and lipoatrophy have been the predominant reasons for stavudine substitutions. In a study in Thailand, 35 HIV-infected adults were switched from stavudine/didanosine to tenofovir/lamivudine. There was an increase in limb fat mass (+0.38 kg, *p*<0.006) and total fat mass (+0.69 kg, *p*<0.02), as measured by DEXA 48 weeks after the regimen change [99]. In addition to improving lipoatrophy, switching from stavudine to tenofovir was associated with increased mtDNA copies (386–1537, *p*<0.001) [142] and decreased total cholesterol, LDL and triglycerides [99,142,143].

Several studies in low-income and middle-income countries have examined the effects of stavudine substitution with zidovudine. In a study in Rwanda among HIV-infected adults with lipodystrophy who were switched to either zidovudine or tenofovir/abacavir, the zidovudine group had significantly greater weight loss three months after substitution, with a mean loss of 1.6 kg (−1.62±4.23 kg) by 12 months. The tenofovir/abacavir group had a stable body weight for the first three months and a small but significant weight increase by 12 months (+0.70±4.18 kg) [144]. No studies to date have evaluated the efficacy of switching to NRTI-sparing regimens in low-income and middle-income countries. In high-income countries, this regimen change has been effective in maintaining HIV viral load suppression and reducing the risk of side-effects, including lipodystrophy, though NRTI-sparing regimens are not commonly used in clinical practice [145,146].

### Pregnant women

There is a major research gap in our understanding of lipodystrophy during pregnancy. Pregnancy is a time of many hormonal and metabolic changes that directly affect the health of the woman and foetus. During pregnancy, there is a transient increase in lipid parameters, which may be exacerbated with NRTI and PI use [147,148]. Further research is warranted to understand how lipodystrophy presents during pregnancy, and the impact of lipodystrophy on maternal and neonatal health. Further understanding of lipid alterations and naturally occurring insulin sensitivity in early trimesters and insulin resistance later in pregnancy [149] should inform ARV treatment options, particularly in resource-limited settings where perinatal HIV infection is common.

## Discussion

ART is the gold standard for HIV/AIDS treatment, based on its demonstrated benefits on disease progression, immune reconstitution and mortality. However, lipodystrophy, lipoatrophy, lipohypertrophy and a mixed syndrome are common metabolic complications of ART, and increases with ART type and duration. There is limited research on ART-related lipodystrophy in low- and middle-income countries, despite accounting for more than 95% of the burden of HIV/AIDS. Of the 90 studies reviewed among adults, only eight were published from five low-income countries and nine from two lower middle-income economies. These studies focused on lipodystrophy prevalence, risk factors and ART side-effects, reflecting the continued use of older toxic ART regimes such as stavudine. Further research is needed to characterize lipodystrophy, its implications and treatment options in lower-income countries.

The prevalence of lipodystrophy is considerable in low- and middle-income countries, with lipoatrophy specifically increasing with type and duration of therapy. In most studies, lipoatrophy developed after the first six months of therapy, particularly with use of stavudine. Despite WHO recommendations, 14 out of 52 developing countries still use stavudine as a first-line HIV therapy, due to its comparatively lower cost and widespread availability [16,114,115]. Therefore, in addition to prevalent cases of lipoatrophy from past use of stavudine, there continue to be incident cases from continued stavudine use in resource-poor settings.

Lipodystrophy, lipoatrophy, lipohypertrophy and a mixed syndrome are all associated with increased risk of cardiometabolic complications, including hypertension, diabetes, insulin resistance and cardiovascular disease. Many studies reviewed highlighted the increased odds of developing metabolic syndrome with the presence of lipodystrophy [150–155]. Newer therapies, such as thiazolidinediones, target not just fat redistribution but also insulin resistance associated with lipodystrophy [156–158]. The incidence of metabolic complications from HAART and/or lipodystrophy are anticipated to increase, given the rapid sale-up of ARVs worldwide, the increasing number and lifespan of HIV-infected patients on long-term ART, and emergence of obesity and non-communicable diseases in settings with extensive HIV burden.

Improved prevention and timely resolution of lipodystrophy could impact health, psychosocial well-being, quality of life and adherence. The stigma associated with HIV in some developing countries is still severe, therefore external fat redistribution changes can significantly negatively impact an individual's quality of life and ART adherence [8,131,134,159].

Lipodystrophy management is integral to HIV care and treatment, and warrants further investigation in low- and middle-income countries.

### Limitations

There are several limitations in the studies reviewed. These include a lack of standard clinical definition of lipodystrophy syndromes, varying assessment methods, limited number of prospective studies to examine the effects of ART on lipodystrophy over time, small sample sizes, different combinations of ARV regimens and limited assessment of other nutritional, immunological and metabolic factors. Some of the aforementioned studies were conducted among HIV-infected patients with failed first-line therapy or in case-control studies where patients were selected based on lipodystrophy or other clinical conditions, which limits generalizability to other HIV-infected populations and settings [30,120]. These limitations constrain the interpretability and comparability of findings across studies.

The lack of standard clinical definition and heterogeneous characterization of lipodystrophy pose challenges to examine the aetiologies and risk factors of lipoatrophy, lipohypertrophy and mixed syndrome. Most studies have examined these conditions together as a combined endpoint and had insufficient sample size to examine risk factors for each condition individually over time. Many studies cited the need for a universal standardized method to diagnose lipodystrophy syndromes, validated in both clinical and field settings, to compare findings across studies [12,36].

Prospective studies are needed to examine the effects of ART on lipodystrophy over a sufficient period of time after ART initiation. Patterns of lipodystrophy are also dynamic and may change due to HIV infection itself, and other potential factors. Some factors that were rarely adjusted for in multivariate analyses include other nutritional deficiencies, micronutrient supplement use, opportunistic infections, other co-infections and access to health care services, which are important covariates that can confound the results of the studies reviewed and limit the ability to interpret findings on the relationships between HIV, ART and lipodystrophy.

### Future research

Further research is needed to better understand fat redistribution syndromes in resource-limited settings. There is inadequate research on lipohypertrophy, a condition that unlike mixed syndrome or lipoatrophy is also found in HIV-uninfected populations. Lipohypertrophy may be linked to increased risk of metabolic abnormalities, as there is strong evidence that links visceral fat accumulation to non-communicable diseases such as diabetes and cardiovascular disease [85,160]. However, there is limited data on risk factors for lipohypertrophy in HIV-infected populations on ART.

In low- and middle-income countries, HIV/AIDS, food insecurity, micronutrient deficiencies, other infectious diseases and underweight co-occur with the emergence of obesity and non-communicable diseases. Despite the extensive burden of malnutrition, few studies take malnutrition into consideration when evaluating lipodystrophy [106]. Further research is needed on the role of nutrition in the aetiology and clinical management of lipodystrophy, including the interplay between nutrition, metabolic disease, lipodystrophy and HIV infection.

## Conclusions

HIV represents a serious threat to global health, and ART is the gold standard for HIV/AIDS care and treatment. However, adverse ART-related outcomes, including lipodystrophy, are common in low- and middle-income countries. This has considerable implications for risk of metabolic diseases, quality of life and adherence. As ART is rapidly scaled up worldwide and early HIV detection, ARV initiation and lifespans of infected individuals increase, the length of ARV exposure and burden of related metabolic complications are also expected to rise in low- and middle-income countries. Comprehensive evidence-based interventions are urgently needed to reduce the burden of HIV and lipodystrophy, and inform clinical management in resource-limited settings.

## Figures and Tables

**Table 1:** Low-income countries

Region	Population	*N*	Study objective	Lipodystrophy, lipoatrophy, lipohypertrophy	Other findings
**AFRICA**					
Prospective cohort					
Benin [33]	HIV+ initiating HAART	79	LD incidence and risk factors of NNRTIs	LD: 30% (11 mo) Mixed: 2.5%, LH: 24%, LA: 9%	Significant weight gain on ART, 13% developed metabolic syndrome
Uganda [107]	HIV+ initiating HAART	76	Anthropometry, total fat, lean mass with AZT		HIV+: increased lean and fat mass; arm, waist, hip and thigh circumferences; and abdomen, subscapular, thigh SFT at 6 mo vs. controls
Cross-sectional					
Ethiopia [154]	HIV+ on HAART >1 y	356	Prevalence and risk factors of LD	LD: 68.3%	LA/LH risk factors: smoking, d4T, >1 y ART
Ethiopia [152]	HIV+ on HAART	313	Prevalence of BFR and metabolic changes	LD: 12.1%	LD risk factors: ART >12 mo
Malawi [165]	HIV+ on d4T for ≥6 mo	203	d4T toxicity and mtDNA/nDNA ratio	LD: 18% (25 mo)	No association between LD and mtDNA/nuclear DNA ratio
**ASIA**					
Cross-sectional					
Cambodia [44]	HIV+ initiating ART	467	Immunovirological ART outcomes, adverse events	LD: 63% (4 y)	d4T adverse events: BFR, dyslipidemia, increased liver enzymes, peripheral neuropathy
Cambodia [166]	HIV+ on ART ≥2 y	346	Prevalence and predictors of non-adherence		Independent predictors of non-adherence: LD- related side-effects, rural residence, HIV disclosure to <2 family members
Retrospective					
Cambodia [32]	HIV+ initiating d4T	2481	Long term toxicity (6 y) with d4T	LA: 0.8% (6 mo), 7% (1 y), 56.1% (3 y), 72.4% (6 y)	d4T Regimen change: 37.1% due to LD Risk Factors: age >40 y, female, CD4 <200 cells/μL

WHO GDP $1,025 or less. ABC: abacavir; ART: antiretroviral therapy; AZT: zidovudine; BFR: body fat redistribution; d4T: stavudine HAART: highly active antiretroviral therapy; HIV: human immunodeficiency virus; LA: lipoatrophy; LD: lipodystrophy; LH: lipohypertrophy; mo: months; SFT: skinfold thickness; TC: total cholesterol; TG: triglycerides; WC: waist circumference; WHR: waist to height ratio; y: years. Prevalence unless otherwise noted.

**Table 2:** Lower middle-income countries

Country	Population	*N*	Study objective	Lipodystrophy, lipoatrophy, lipohypertrophy**	Other findings
**AFRICA**					
Cross-sectional					
Cameroon [12,36]	HIV+ adults on d4T or AZT ≥6 mo	249	LD prevalence, effect of lower d4T dose on LD	Mild-severe LD: 60% Moderate-severe LD: 18%	Odds of LD: higher in d4t vs. AZT and group on 40 mg d4T switched to 30 mg vs. 30 mg only
**ASIA**					
Prospective cohort					
India [167]	HIV+ initiating NVP ART	190	Effects of NVP on weight, BMI, body composition		Weight changes: At 6 mo, 22% lost mean 3.6 kg, 19% stable, 59% gained mean 6 kg. No differences in WHR ratio, BFR, or CD4 counts.
India [30]	HIV+≥12 mo of RTV-boosted PI	91	Safety, tolerability, efficacy of PI vs. NNRTI	LD incidence: 10.4% in 1 y after NNRTI to PI regimen change	Adverse effects after switch: LD, nausea, peripheral neuropathy
India [125]	HIV+ initiating ART	68	Metabolic and BFR changes with ART		6 mo on ART, 19.1% had new-onset metabolic syndrome; total body fat, body fat mass, lean body mass, SFT significantly increased
India [31]	Malnourished HIV+ adults initiating ART	34	Clinical markers used to monitor BFR	LA: 26% LH: 24	More body weight changes diagnosed than self-reported; 28% men, 75% women
Cross-sectional					
India [51]	HIV+ on ART, HIV+ ART naïve, and HIV−	363	LD prevalence in rural patients receiving ART	LD: 60.7%, LH: 22.7%, LA: 51.1%, mixed: 22.7%	Risk factors for LD/LA: female, ART duration; BMI: HIV−>HIV+ART+>HIV+ART−
India [161]	HIV+ adults on 2-drug ART or HAART	286	Long-term metabolic and BFR changes on ART	LA: 2.8% (21 mo)	37.5% with LA received 2 drug regimen, 50% received HAART, 12.5% both
India [61]	HIV+ men, on ART vs. ART naïve	121	Objective LD definition w/regional fat mass ratios	LD: 54.3% *via* trunk fat/lower limb fat mass ratio >2.28	Clinical diagnosis of LD with many FP/FN results. Triceps SFT/Abd SFT: poor specificity
India [134]	HIV+ adults on HAART	50	QoL among HIV+ adults with and without LD	LA: 42% (3.71 y)	Significant differences in financial (*p*<0.045) and disclosure (*p*<0.023) concerns with LD

GDP per capita: $1026 to $4,035. ABC: abacavir; ART: antiretroviral therapy; AZT: zidovudine; BFR: body fat redistribution; d4T: stavudine; FN, false negative; FP, false positive; HAART: highly active antiretroviral therapy; HIV: Human Immunodeficiency Virus; LA: lipoatrophy; LD: lipodystrophy; LH: lipohypertrophy; mo: months; NVP: nevirapine; PI: protease inhibitor; QoL: quality of life; RTV: ritonavir; SFT: skinfold thickness; TC: total cholesterol; TDF: tenofovir; TG: triglycerides; WC: waist circumference; WHR: waist to height ratio; ZDV: zidovudine; y: year. Prevalence unless otherwise noted.

**Table 3:** Upper middle-income countries

Country	Population	*N*	Study objective	Lipodystrophy, lipoatrophy, lipohypertrophy	Other findings
**AFRICA**					
Prospective cohort					
Botswana [168]	HIV+ subtype C, initiating ART	650	Efficacy of ZDV/ddI/3TC switched from d4T/3TC	Treatment-modifying LD: 36% (2 y)	6.2 kg and 3 kg weight increase from baseline in HIV wasting and HIV non-wasting, respectively
Botswana [43]	HIV+ initiating d4T, NVP	2190	Incidence, risk factors for d4T and NVP toxicity	LA prevalence requiring regimen change: 7.2% (18 mo)	LA risk factors: female sex, 40 mg d4T
Botswana [34]	HIV+ on ART (100 LD, 50 non-LD), 50 HIV−	571	Prevalence of LD and HAART toxicity	LD: 34% (LA: 9%, LH: 19%, mixed: 72%), 18 mo: 69.6%	LD risk factors: urban residence, ART duration; LD associated with significantly higher TC, WHR
Rwanda [11]	HIV+ adults on ART for >1 y	409	Prevalence and risk factors of LD	LD: 34.3% (16 mo), mixed: 19.6%, LH: 4.9%, LA: 9.8%	LA risk factors: recent onset/faster rate weight loss, 3x higher LA with d4T vs. AZT, female sex
Rwanda [144]	HIV+ requiring d4T substitution for LA	114	Weight after d4T switch to ZDV or TDF/ABC		Switch to ZDV: progressive weight loss (significant at 2 and 12 mo); Switch to TDF/ABC: stable body weight, increased slightly after 3 mo
Rwanda [133,135]	HIV+ with and without BFR, HIV−	100	Weight and QoL after 6 mo of exercise therapy		Greater decline in WC, WHR; improved emotional stress, body image, self-esteem psychological well-being, social relationships, and independence in exercise group
South Africa [42]	HIV+ initiating ART	9040	Long term incidence of d4T toxicity, risk factors	LA incidence: 7.3%/4.6% after 23.1 mo of d4T/non-d4T ART	LA risk factors: d4t and female sex
South Africa [116]	HIV+ initiating ART	3910	Efficacy and safety of 30 mg vs. 40 mg of d4T	LA incidence: <2% (12 mo)	No evidence of impaired viral suppression with 30 mg d4T; LA: Increased odds with 40 mg d4T
South Africa [4]	HIV-infected on HAART	2815	ART toxicity and reasons for regimen change	LD prevalence: 8.3% (14.9 mo)	HAART adverse effects: LD, lactic acidosis
South Africa [48]	HIV+ initiating HAART	2679	ART toxicity and reasons for regimen change	LD prevalence requiring d4T substitution: 9% (3 y)	LD risk factors: female sex, ART ≥6 mo, d4T regimen change: 21% (9% due to LD)
South Africa [35]	HIV+ initiating ART	230	Changes in perception and anthropometry	LD prevalence: 35% (12 mo)	Significant discrepancy between BMI and patient's perception of weight
South Africa [49]	HIV+ initiating d4T ART	42	Changes in BFR and metabolic factors on d4T	LD: 42.9% (20.5 mo) mixed: 33%, LH: 9.5%, LA: 9.5%	LD risk factors: greater triceps and iliac crest SFT at baseline
Retrospective					
Rwanda [106]	HIV+ initiating d4T	705	Weight change with LA and efficacy of d4T	LA requiring regimen change: 12.2% (2 y)	Rapid weight increase in first 6–12 mo of ART, progressive decline in weight year 2
Cross-sectional					
Rwanda [21]	HIV+ ART naïve and HIV− controls	821	BFR in HIV− and HIV+, ART naïve women		Mean body weight, BMI (19% HIV+, 26% HIV−), total body fat by BIA, WHRs similar in both groups; no associations between CD4 counts and body composition
Rwanda [8,159]	HIV+BFR+ and HIV+ BFR−	100	Relationship between BFR and QoL		HIV+BFR+ vs. HIV+BFR−: former with larger WHR, less satisfied with self-esteem, social life, body appearance, more emotional stress, ashamed in public places
Senegal [12]	HIV+ on HAART 4–9 y and HIV− controls	180	Prevalence, risk factors, metabolic disorders	LD: 65%, mild: 33.9%, severe: 31.1%; 2nd LD definition: 49.5%	Lower BMI, skinfolds and greater WHRs and trunk/arm skinfold ratios on HAART
Senegal [169]	HIV+ on HAART	20	Perceptions of LD	LD: 31.1% (9–16 y), LA: 60%, LH: 25%, Combined: 15%	Most unable to recognize changes as LD. LH tolerated better; excess weight sign of wealth
Senegal [170]	HIV+ women, ART naïve, d4T/AZT, LPV/r	744	Body composition (DEXA) of d4T/AZT vs. LPV/r		D4T/AZT or LPV/r: more central fat mass (*p*<0.01) and less leg fat mass (*p*<0.01) than ART naïve. D4T: WHR greater, calf skinfold lower (*p*<0.033) with increasing d4T time
Senegal [171]	HIV+ exposed to thymidine analogue	171	mtDNA haplotypes, metabolic changes		No significant associations between mtDNA haplogroups and lipoatrophy
South Africa [172]	HIV+ on HAART	479	Demand for surgical LA correction	LD: 11.7%	5.9% considered stopping therapy due to LD, 47% consider surgery to correct LA/LD
South Africa [173]	HIV+, ART naïve women on ART	83	VL, immune activation, adipose tissue		High VL women had a lower BMI, subcutaneous abdominal fat, WC, trunk fat measurements with DEXA vs. low VL group
South Africa [174]	HIV+ ART naïve, ART with LD+, ART LD−	39	PET glucose uptake by fat and muscle		Standardized uptake values (SUV): Higher in HAART patients with LD; SUV values for subcutaneous fat correlated with treatment duration and CD4 count
**ASIA**					
Randomized trials					
China [175]	HIV+ on d4T vs. switch to AZT after 24 weeks	199	Effects of switching from d4T to AZT after 24 weeks	LD: 18.4%, d4T to AZT group; LD: 0.6%, AZT only group	
Prospective cohort					
China [24]	AIDS+, ART naïve adults	103	Efficacy/side-effects of HAART in AIDS patients	LD: 9.7% (12 mo)	HAART side-effects: peripheral neuropathy, gastrointestinal disorders, LD
China [25]	HIV+ adults initiating ART	57	Long-term ART outcomes	LD: 10.5% (72 mo)	44% changed from d4T to ddI (24 mo); 26.3% with elevated cholesterol and TG (72 mo)
China [26]	HIV+, ART naïve adults initiating ART	52	Adipocytokine and adipose distribution in HIV/LD	LD incidence: 2% (12 mo), 9.6% (18 mo), 52% (30 mo)	Adiponectin incresed (6 mo) then decreased (18–30 mo) in LD+ group, no change in LD− group
Thailand [27]	HIV+ adults on HAART	829	Genetic polymorphisms correlated with LA/LD	LA or LD: 32.6% (4 y)	LA or LD risk factors: female sex, d4T (compared to AZT), Fas-670-AA
Thailand [28]	HIV+ initiating d4T/NVP	152	Efficacy and adverse effects of d4T/3TC/NVP	LD: 35.5% (3 y)	Median % body weight increased 8.1% (1 y) then plateaued until end of study
Thailand [29]	HIV+ initiating d4T/NVP	83	Efficacy and adverse effects of d4T/3TC/NVP	LD: 16.8% (2 y)	LD risk factors: low baseline body weight (≤50 kg), female sex, duration of treatment
Thailand [120]	HIV+, NRTIs to RTV boosted IDV/EFV	61	Metabolic disturbances and changes in BFR		VAT mass increased by 20% (96 weeks) after switch; 4.1 and 15.9% increase in TC and TG, respectively, with 1 kg reduction in total limb fat mass at baseline
Thailand [121]	HIV+, NRTI to NRTI-sparing regimens	60	Effect of switch: NRTI to NRTI-sparing ART	All had LD	DEXA: mean total limb fat increase, mean lean limb mass loss (48 weeks) after switch
Thailand [99]	HIV+ 48 weeks after d4t/ddI to TDF/3TC	35	Effect of medication change on mtDNA	LD: 54%	After switch, mtDNA content rose significantly, lipid factors reduced; reversal of LA on DEXA
Cross-sectional					
Thailand [155]	71% HIV+ on ART, 29% ART naïve	580	Risk of metabolic syndrome with LD	LD: 45.2%, LA: 41.9%, LH: 28.1% (2.6 y)	LD+: OR 1.8 (95% CI 1.0–3.0, *p*=0.032) of developing metabolic syndrome
Thailand [130]	HIV+ 33% treatment naïve, 77% ART	278	Prevalence, clinical characteristics of LD	LD: 21% in ART (43 mo)	LD associations: higher CD4 counts and lower viral loads; 93% had 1+ metabolic abnormality
Thailand [62]	HIV+ART naïve, on PI ART, or non-PI ART	247	Prevalence of BFR and metabolic changes	LA: 21% temporal, 28% facial LH: 53% PI, 12.15% non-PI	ART treated: higher WC and WHR LA risk factors: Use of d4T and indinavir
Thailand [117]	HIV+ >15 y old, exposed to d4T	103	LD risk factors	Absent to mild LD: 47% Moderate to severe LD: 53%	Increased odds of LD: HLA-B*4001 status, 2% increase of LD for every month of d4T
Thailand [50]	HIV+ initiating ART	56	Prevalence, clinical, risk factors of LD	LD: 66.1%; mixed 26.8%, LA 35.7%, LH 3.6%	LD risk factors: undetectable HIV RNA, d4T
**CENTRAL/SOUTH AMERICA**					
Prospective cohort					
Brazil [37]	HIV+ on HAART	332	Identify risk factors for LD in HIV/AIDS	LD: 54.8% (5.7 y)	LD: higher BMI (women), lower BMI (men), LD risk factors: d4T >3 y, HAART >4 y
Brazil [54]	HIV+LD+ and HIV− with PMMA fillers	266	Determine efficacy/safety of PMMA as facial filler		90% of subjects were satisfied with treatment, significant improvements in self-esteem; Adverse effects all transient and resolved spontaneously
Brazil [176]	HIV+ LD+ on HAART>1 year	187	Evaluate body fat content based on ART duration		LD+ had 11 mm of additional abdominal fat after 1 y of ARVs vs. LD− Women had greater peripheral fat and men had 7.2 mm more visceral fat
Brazil [177]	HIV+LD+ with/without fibric acid therapy	53	Compare changes in lipid metabolism in LD adults		No change in metabolic parameters at 6 mo post-nutritional counseling
Brazil [58]	HIV+ LA+, ART naive	44	PMMA treatment on HIV disease progression		Non-significant increase in patients with undetectable viral load (*p*=0.67) and significant increase in CD4 count (*p*=0.02) after PMMA treatment
Brazil [55]	AIDS+ with PMMA filling for LA	49	Evaluate the use of PMMA to treat LA		Most patients on d4T or ZDV; side effects infrequent; 85.7% of patients satisfied after >12 mo with PMMA
Brazil [178]	HIV+ LD+ on HAART for ≥18 mo	24	Metformin's effects on HIV+LD+ adults		Mean weight loss: 2.2% of initial body weight (3 mo), 3.8% (6 mo), BMI reduction, WHR reduction, CT: total abdominal tissue reduced by 10.3% (6 mo)
Brazil [56]	HIV+ with facial LA	20	Using Index for facial LA (ILA) to diagnose LA		Good to excellent response to treatment for moderate to severe facial LA; short-term side effects resolved <1 week and all within 6 mo
Randomized trial					
Brazil [140]	HIV+ on HAART for ≤12 mo	50	Nutritional counseling on diet to prevent BFR		No differences between intervention (dietary counseling) and control; both groups showed an increase in SSF from 1–8 mo (*p*<0.001)
Brazil [179]	HIV+ LA+: NRTI-sparing for >24 weeks	71	Rosiglitazone-induced fat recovery in LA		Limb fat (DEXA) increased significantly (448 g vs. 153 g) in LA group treated with rosiglitazone vs. placebo; facial LA scores changed significantly
Brazil [59]	HIV+ on ART eligible for PMMA for LA	40	Effects and impact of facial LA on patient QoL		Slight improvement in medical outcomes, self-esteem, and Beck depression scale in PMMA group
Brazil [137]	HIV+ LD+ HL+ adults on HAART >6 mo	30	Evaluate the effect of exercise therapy on LD		Weight, BMI,% BF decreased similarly in both exercise/diet and diet groups; peak oxygen uptake improved only in exercise/diet
Cross-sectional					
Argentina [180]	HIV+ART naïve, ART LD+/LD−	101	Markers of endothelial function in HIV+ adults		LD group: highest TG, sVCAM-1 correlated with vWF (endothelial activation markers), % arm fat, CD4 T-cell counts, TG, ART naïve group: highest sVCAM-1
Argentina [74]	HIV+ on HAART	72	Congruency between methods measuring BFR		Questionnaire A vs. B: 86%/49% with BFR; Clinical criteria A vs. B: 48%/24% Best assessment: DEXA ratio between trunk fat content and the leg fat content
Brazil [181]	HIV/AIDS+	958	LD and HTN prevalence	LD: 46.1% (HTN) LD: 47.7% (pre-HTN)	Increased risk of HTN: age >40 y, male sex, BMI >25, TG >150 mg/dL
Brazil [182]	HIV+	819	Metabolic disturbances and LD prevalence	LD: 38.5%; LA: 45%, mixed: 42.9%, LH: 12.1%	Metabolic parameters: 86% had ≥1 metabolic disturbance (low HDL, high TG)
Brazil [183]	HIV+ receiving HAART	620	ART on lipid profile pre- and post-HAART		Prevalence of DL/LD after HAART was 32.4% 30.1 mo vs. 11.3% before HAART Higher prevalence of DL/LD with PI based regimen and d4T (*p*=0.006)
Brazil [184]	HIV+ on HAART >1 y	614	Genetic SNPs in estrogen receptor-a and -b in LD		LA more common in men, LH more common in women (35.6% of all LD in women) LA risk factors: age, European ancestry, PI use, AA homozygotes for ESR2 rs3020450
Brazil [122]	HIV/AIDS adolescents on HAART	457	Analysis of patient self-perception of BFR		64.3% reported perception of body changes (15% peripheral loss, 27% central gain); Perceived body changes significantly associated with duration of PI, NRTI, not NNRTI
Brazil [38]	HIV+ on HAART	410	Association between 4 polymorphisms and LD	Prevalence of LD: 53.4%	No difference in genotype frequencies (LD/LD−); Adiponectin higher in specific alleles in LD
Brazil [185]	HIV+LD+, HIV+LD−HIV−LD−	300	IL-18 and IFN-y gene polymorphisms (LD/LD−)		LD: increased mRNA expression of inflammatory cytokines TNF, IL-6, IL-8; Increased odds of LD: IL-18-607A, Decreased odds: IL-18-607C; IFN-y: no difference (LD/LD−)
Brazil [186]	HIV+	256	Estimate hyperApoB prevalence, CV risk		Self-reported LD not associated with high apoB, prevalence of hyperApoB: 32.4% Hypercholesterolemia: 32%; TG: 47%; low HDL 47.6%, high LDL 47.4%
Brazil [136]	HIV/AIDS+ on HAART for ≥3 mo	220	Physical activity and central fat relationship		3 physical activity questionnaires: occupation, leisure sports, locomotion; significant negative correlation for LTPA with CSF
Brazil [39]	HIV or AIDS+ (96%)	180	Assess LD prevalence and characteristics of LD	Women: LH: 43%, mixed: 40%; men: LA: 34%, mixed: 34%, LH: 32%	Risk factors: female sex, AIDS duration >8 y
Brazil [82]	HIV+LD+ and HIV+LD−	179	U/S vs. clinical signs used to diagnose BFR		LD risk factors: age, greater duration to ART; U/S predictors of LD: facial, subq abd, visceral body compartments; ROC curves: sensitivity 69.1%, specificity 94.9%
Brazil [187]	HIV+ART, HIV+ART naïve adults	160	Compare intra-abdominal fat thickness by U/S		No significant difference in subcutaneous adipose tissue between groups; positive correlation between intra-abdominal fat thickness and plasma TG, TC, glucose
Brazil [162]	HIV+LD+; HIV+LD− controls	139	Different morphological alterations from LD	LA: 38.3% (25% facial); LH: 15% (8.3% abdomen); mixed: 46.7% (10% abdomen, face, UL, LL, 6.6% abdomen, face)	
Brazil [188]	HIV+LD+, HIV+LD−	117	Frequency of TNF-a-e microsatellites in HIV		TNF-308 G associated with OR: 28.40 of developing LD, protective effect of TNF-308G: 0.39, no significant association with TNF microsatellite presence polymorphisms
Brazil [189]	HIV+LD+, HIV+LD−	69	LD+ and immune status correlation		Reduced CD4 T-cells: lower antibody production, lower anti-mLDL levels LD correlated with increased mLDL, decreased anti-mLDL antibodies
Brazil [190]	HIV+LD+, HIV+LD−healthy controls (men)	44	Assess HIV-LD energy expenditure		HIV+LD+ vs. LD−: REE per kg lean mass significantly greater, body fat% lower, especially in leg; all HIV+: increased carbohydrate oxidation, lower lipid oxidation
Brazil [191]	AIDS+ patients on HAART	74	Prevalence of LD after ≥5 y on HAART	Self-reported LD: 51.4%, anthropometric: 66.2%, objective verification: 32.4%; LA: 62.5%, LH: 8.3%, mixed: 29.2%	
Brazil [70]	HIV+LD+, HIV+ LD−, controls (men)	44	Compare different methods to diagnose BFR		BIA positively and significantly correlated with DEXA for MUAC and lean mass; SFT values correlated with DEXA only in controls
Brazil [40]	HIV+ on HAART	42	Assess physical activity in LD in HIV+ adults	LD: 42.9%	Active individuals had a 79% less chance of developing LD versus sedentary individuals
Retrospective					
Brazil [69]	HIV+ (PI: 47 NNRTI: 37)	102	Factors associated with HIV related LD	LD: 59.3%	LD associated with increase in TC, not HDL nor TG
Brazil [192]	HIV+ starting on HAART	233	Adverse effects of d4T	LD: 6.9%	
Chile [41]	HIV+ on HAART for ≥10 y	121	Clinical outcomes after 10 y of HAART		Average of 3.5 different ART regimens in 10 y due to drug toxicity (35%), allergy (9.2%) dyslipidemia (5.6%), lipodystrophy (5%)

GDP per capita: $4,036–12,474. 3TC: lamivudine; ABC: abacavir; AIDS: acquired autoimmune deficiency syndrome; apoB: apolipoprotein B; ART: antiretroviral therapy; AZT: zidovudine; BFR: body fat redistribution; BMI: body mass index; CSF: central subcutaneous fat; CV: cardiovascular; d4T: stavudine; ddI: didanosine; DEXA: dual energy X-ray absorptiometry; EFV: efavirenz; HAART: highly active antiretroviral therapy; HIV: human immunodeficiency virus; IDV: indinavir; LA: lipoatrophy; LD: lipodystrophy; LH: lipohypertrophy; LTPA: leisure time physical activity; mtDNA: mitochondrial DNA; mo: months; MUAC: mid upper arm circumference; NVP: nevirapine; PET: positron emission tomography; PI: protease inhibitor; PMMA: polymethylmethacrylate; QoL: quality of life; REE: resting energy expenditure; ROC: receiver operating curve; SFT: skinfold thickness; TC: total cholesterol; TDF: tenofovir; TG: triglycerides; TNF: tumor necrosis factor; U/S: ultrasound; VAT: visceral adipose tissue; VL: viral load; WC: waist circumference; WHR: waist to height ratio; ZDV: zidovudine; y: year. Prevalence unless otherwise noted.
